# Clinical Use of Bayesian Model-Informed Precision Dosing in Routine Practice: A Focused Systematic Review

**DOI:** 10.3390/jcm15103838

**Published:** 2026-05-15

**Authors:** Wael A. Alghamdi

**Affiliations:** Department of Clinical Pharmacy, College of Pharmacy, King Khalid University, Abha 62223, Saudi Arabia; walghamdi@kku.edu.sa

**Keywords:** Bayesian dosing, model-informed precision dosing, therapeutic drug monitoring, routine clinical practice, software, pharmacokinetics

## Abstract

**Background:** Bayesian model-informed precision dosing (MIPD) is increasingly used to individualize drug therapy; therefore, this review aimed to identify and characterize its implementation in routine clinical practice. **Methods:** A focused systematic review was conducted. Web of Science Core Collection and PubMed were searched from inception to February 2026. Eligible studies were original research articles evaluating Bayesian MIPD in routine clinical practice using software platforms that supported dosing decisions. Data were synthesized descriptively. No formal risk-of-bias assessment was performed due to heterogeneity in study design. **Results:** Fifteen studies met the inclusion criteria. Anti-infective therapy predominated, particularly vancomycin (*n* = 11), with additional studies involving busulfan, mycophenolate mofetil, amikacin, and tobramycin. Commonly reported software platforms included InsightRx (*n* = 6) and DoseMeRx (*n* = 4), along with Abbottbase, NextDose, and ISBA. MIPD was mainly applied with therapeutic drug monitoring, reflecting predominant *a posteriori* use in routine care. Across studies, implementation was associated with improved pharmacokinetic target attainment, while a subset reported clinical benefits, including reduced nephrotoxicity and favorable effectiveness-related outcomes. Pharmacist involvement was commonly described. **Conclusions:** Published evidence indicates that Bayesian MIPD is being implemented in routine clinical settings, but current published experience is dominated by vancomycin-focused studies. Although the evidence base remains limited, it has grown since 2020 and suggests that software-supported Bayesian dosing can improve pharmacokinetic target attainment and may support better clinical outcomes.

## 1. Introduction

Interindividual variability in drug exposure remains a major challenge in clinical pharmacotherapy, particularly for drugs with narrow therapeutic indices or high pharmacokinetic variability. Conventional dosing approaches may not adequately account for interindividual variability and patient-specific factors, which can contribute to underexposure or toxicity [1]. As a result, there is increasing interest in strategies that enable individualized dosing to optimize treatment outcomes.

Bayesian model-informed precision dosing (MIPD) has been increasingly recognized as a framework to individualize drug therapy by integrating patient-specific data with population pharmacokinetic models [2]. By combining prior knowledge with individual patient characteristics and measured drug concentrations (when available), Bayesian approaches allow dynamic estimation of drug exposure and support dose optimization tailored to the individual patient. In recent years, the development of software platforms has facilitated the clinical application of Bayesian dosing. These tools enable implementation of MIPD in routine care by providing estimation of pharmacokinetic parameters and dosing recommendations [3]. Bayesian software has demonstrated good predictive performance in clinical datasets and has been shown to support achievement of target drug exposures, particularly in antimicrobial therapy [4].

Clinical use of MIPD has been increasingly reported across different therapeutic areas, including anti-infective therapy, transplantation, and critical care [5,6,7]. In particular, its integration with therapeutic drug monitoring (TDM) has enabled more precise estimation of drug exposure and improved dose individualization in routine practice.

Despite this growing interest, a key gap remains between the extensive body of literature evaluating MIPD and the relatively limited number of studies that document and evaluate its implementation in routine clinical practice. Much of the existing evidence is based on retrospective comparisons, methodological developments, or evaluation of predicted performance, rather than real-world application in clinical workflows [8,9,10,11]. Moreover, the absence of published studies does not necessarily indicate the absence of clinical use but rather highlights limited reporting of implementation in routine care.

Therefore, this systematic review aimed to identify and characterize the routine clinical implementation of Bayesian model-informed precision dosing, with a particular focus on software-supported applications in real-world clinical practice.

## 2. Methods

### 2.1. Study Design

This study was conducted as a systematic review to identify and characterize the clinical implementation of MIPD using Bayesian approaches in routine practice. The review was performed in accordance with the Preferred Reporting Items for Systematic Reviews and Meta-Analyses (PRISMA) 2020 guidelines (Supplementary S1. PRISMA checklist) [12]. This review was not prospectively registered, and no formal protocol was prepared.

### 2.2. Literature Search

A systematic literature search was performed in Web of Science Core Collection and PubMed from database inception to February 2026. The search was conducted in March 2026 and included all records indexed up to 28 February 2026. The search strategy combined terms related to Bayesian dosing, model-informed precision dosing, therapeutic drug monitoring, and clinical implementation of software or decision support tools.

Key search terms included combinations of “Bayesian,” “precision dosing,” “model-informed precision dosing,” “therapeutic drug monitoring,” “individualized dosing,” and “dose optimization,” along with terms related to software and implementation (e.g., “clinical decision support,” “software,” “platform”) and specific platforms (e.g., DoseMeRx, InsightRx, MwPharm, BestDose, TDMx, NextDose, Tucuxi, PrecisePK). For Web of Science, searches were performed using topic fields, while PubMed searches were conducted using title and abstract fields. Duplicate records were removed prior to screening. Non-original publications, including review articles, conference abstracts or proceedings, and editorial material, were excluded during the initial screening process. Conference abstracts and proceedings were excluded because they generally lacked sufficient methodological and implementation detail for reliable assessment and data extraction. The full search strategies are provided in the Supplementary S2.

### 2.3. Eligibility Criteria and Study Selection

Studies were eligible for inclusion if they were original research articles evaluating the use of Bayesian MIPD in routine clinical practice, involving software tools or platforms that supported dosing decisions in clinical care. Studies were excluded if MIPD was applied exclusively in trial or research settings without routine clinical implementation, if Bayesian methods were not used, or if the study was limited to simulation or model validation without clinical application. Studies focused solely on software or tool development or description without clinical use were also excluded. In addition, review articles, conference abstracts or proceedings, and editorial materials were excluded.

**Figure 1:**
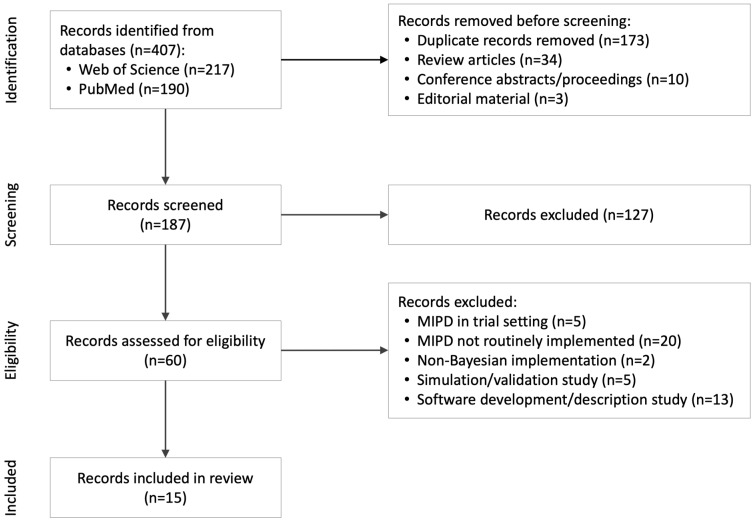
PRISMA flow diagram of study selection.

Study selection was performed by a single reviewer in two stages, consisting of title and abstract screening followed by full-text assessment for eligibility. A total of 187 records were screened, of which 60 full-text articles were assessed. Fifteen studies met the inclusion criteria. The focused scope of the review and predefined eligibility criteria supported consistent application of study selection decisions. The study selection process is summarized in the PRISMA flow diagram (Figure 1).

### 2.4. Data Extraction and Synthesis

Data were extracted from the included studies based on predefined variables, including therapeutic area, drug name, software platform, patient population, pharmacist involvement, outcome domains, study conclusions, country, and publication year.

Given the heterogeneity in study designs, populations, and reported outcomes, a qualitative descriptive synthesis was performed. Findings were summarized and compared across studies, with emphasis on patterns of clinical implementation, software use, and reported outcomes. No quantitative meta-analysis was conducted. A formal risk of bias assessment was not performed because the included studies were heterogeneous in design and primarily descriptive in nature, focusing on implementation rather than comparative effectiveness.

## 3. Results

### 3.1. Study Selection

A total of 407 records were identified through database searching. After removal of duplicates and non-original publications, 187 records were screened by title and abstract. Of these, 60 full-text articles were assessed for eligibility, and 15 studies met the inclusion criteria. The study selection process is summarized in the PRISMA flow diagram (Figure 1). The majority of excluded full-text articles retrospectively compared MIPD with existing non-MIPD practice, rather than reporting implementation in routine clinical care.

### 3.2. Characteristics of Included Studies

The 15 included studies evaluated the clinical use of Bayesian MIPD across a range of therapeutic areas, with the majority focusing on anti-infective agents, particularly vancomycin (*n* = 11). Other drugs evaluated included amikacin (*n* = 1) and tobramycin (*n* = 1). Non–anti-infective applications included busulfan in hematopoietic cell transplantation (*n* = 1) and mycophenolate mofetil in solid organ transplantation (*n* = 1).

Most studies were conducted in the United States, with additional studies from Australia, New Zealand, France, and South Korea. Study populations varied and included both adult and pediatric patients, with several studies focusing on specialized populations such as neonates, critically ill patients, and individuals with cystic fibrosis.

A variety of software platforms were used to support Bayesian dosing, with commonly reported tools including InsightRx (*n* = 6) and DoseMeRx (*n* = 4), alongside other platforms such as Abbottbase (*n* = *2*), NextDose (*n* = 1), and ISBA (*n* = 1). One study used both Abbottbase and DoseMeRx, resulting in a total platform count exceeding the number of included studies. In two studies, the specific software platform was not reported. Across studies, MIPD was applied within routine clinical workflows to guide individualized dosing decisions, often in the context of therapeutic drug monitoring. Pharmacist involvement was commonly reported in the implementation and application of MIPD across studies. Detailed characteristics of the included studies are summarized in Table 1.

### 3.3. Implementation and Outcomes of MIPD

Across the included studies, MIPD was integrated into routine clinical practice to support individualized dosing decisions, most commonly in conjunction with TDM. MIPD was primarily used for dose adjustment following drug concentration measurements (*a posteriori*), reflecting its role in guiding TDM and dose optimization in clinical practice.

Software-supported Bayesian dosing enabled estimation of drug exposure, most frequently expressed as area under the concentration-time curve (AUC) and facilitated achievement of target exposure ranges. This finding is consistent with the predominance of vancomycin-focused studies, for which AUC is the recommended pharmacokinetic exposure parameter. Most studies assessing AUC target attainment reported improved achievement of therapeutic exposure targets, particularly in vancomycin studies, although some studies used AUC primarily for exposure comparison or agreement analysis rather than target attainment assessment.

In addition to pharmacokinetic outcomes, clinical outcomes were reported in a subset of studies. These included reduced incidence of nephrotoxicity, particularly acute kidney injury, as well as measures of clinical effectiveness. Some studies also highlighted improvements in dosing management, including earlier target attainment, reduced need for dose adjustments, and decreased TDM burden. Pharmacist involvement was frequently described in the implementation and application of MIPD, including roles in dose individualization, interpretation of Bayesian estimates, and integration of dosing recommendations into clinical workflows.

Overall, the findings suggest that implementation of Bayesian MIPD in routine clinical care supports more precise and individualized dosing, with improvements in pharmacokinetic target attainment and potential benefits for clinical outcomes and dosing management.

## 4. Discussion

**Figure 2:**
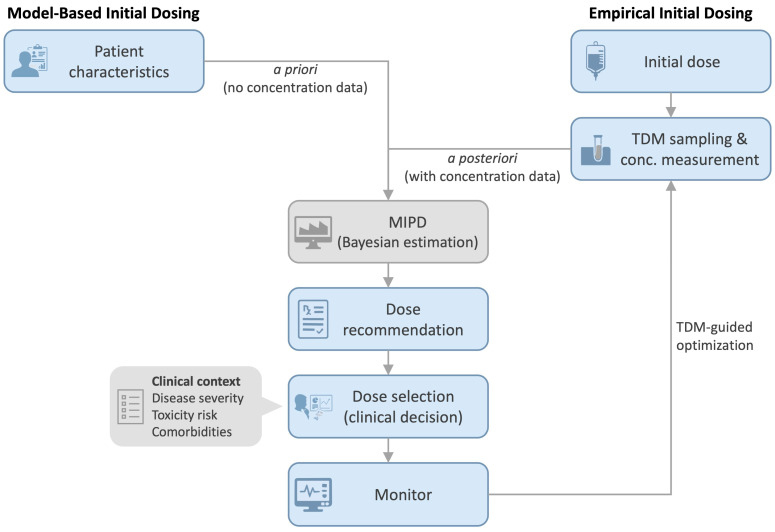
Clinical workflow of model-informed precision dosing (MIPD) using *a priori* and *a posteriori* Bayesian approaches. Model-based initial dosing (*a priori*) uses patient characteristics and population pharmacokinetic models to estimate drug exposure in the absence of concentration data. In contrast, empirical initial dosing is followed by TDM sampling, enabling *a posteriori* Bayesian estimation incorporating measured drug concentrations. Both pathways converge within the MIPD framework to generate individualized dose recommendations. Final dose selection is guided by clinical judgment, informed by patient-specific factors such as disease severity, toxicity risk, and comorbidities. Ongoing monitoring with TDM supports continuous model updating and dose optimization.

The included studies demonstrate that Bayesian MIPD has been implemented in routine clinical practice across a range of settings, predominantly in conjunction with TDM [5,6,7,13,14,15,16,17,18,19,20,21,22,23,24]. In these studies, MIPD was integrated into clinical workflows to support individualized dosing decisions, typically through dedicated software platforms and, in some cases, embedded clinical decision support tools within electronic health records (Figure 2). Pharmacist involvement was commonly reported, with roles including dose individualization, interpretation of Bayesian estimates, and integration of dosing recommendations into patient care processes. These findings suggest that, when implemented, MIPD functions as a practical tool to support clinical decision-making rather than solely as a theoretical or research-based approach.

This systematic review identified a limited but growing body of literature describing the real-world clinical implementation of Bayesian MIPD. The included studies were heavily focused on anti-infective therapy, particularly vancomycin (*n* = 11), and the reported pharmacokinetic and clinical outcomes were therefore largely driven by vancomycin-focused applications, with fewer studies in other therapeutic areas such as hematopoietic cell transplantation (busulfan) and solid organ transplantation (mycophenolate mofetil). As a result, the reported pharmacokinetic and clinical outcomes were largely driven by vancomycin-focused applications, where AUC-guided dosing and nephrotoxicity outcomes are well established in clinical practice and guidelines [25]. Across studies, software-supported Bayesian dosing was consistently used to estimate drug exposure and guide dose optimization, most commonly in the context of TDM.

A key finding of this review is the marked gap between the extensive body of literature on MIPD and the relatively limited number of studies that document and evaluate its use in routine clinical practice. During full-text screening, the majority of excluded studies retrospectively compared MIPD with existing non-MIPD practice or retrospectively evaluated predicted performance of Bayesian dosing approaches, rather than reporting real-world implementation. This highlights that, although MIPD has been extensively studied through retrospective comparisons with existing clinical practice, as well as through retrospective evaluation of its predicted performance, its translation into published real-world clinical practice remains less frequently reported [8,9,10,11]. Importantly, the absence of published studies does not necessarily indicate the absence of clinical use in practice. It is likely that MIPD is implemented in practice in settings where formal evaluation or publication has not been undertaken. Nevertheless, the available evidence suggests that systematic reporting of routine clinical implementation is still emerging.

Conference proceedings and meeting abstracts also suggest broader activity in Bayesian dosing software than is captured by the small number of included full-text studies [26,27,28,29,30]. However, the available records appear to focus mainly on software presentations, retrospective predictive-performance analyses, feasibility studies, or pharmacokinetic model validation rather than evaluation of routine clinical implementation. For example, a meeting abstract using routine clinical care data evaluated whether prior Bayesian estimates improved prediction in repeat vancomycin courses, illustrating the ongoing real-world application of Bayesian dosing software in a study centered on predictive performance [29]. This further highlights the distinction between the broader Bayesian dosing literature and the smaller body of published studies specifically documenting routine clinical use.

Notably, all included studies were published from 2020 onward, indicating that documentation of routine clinical use of MIPD is a relatively recent development. This temporal pattern suggests increasing adoption and recognition of MIPD in clinical practice but also underscores the need for more comprehensive and systematic evaluation of its real-world use. Although MIPD can theoretically be applied both *a priori* and *a posteriori*, the included studies primarily reflect its use in conjunction with TDM, highlighting the predominance of *a posteriori* applications in routine clinical care. Figure 2 provides a conceptual overview of how these two approaches converge within software-supported Bayesian dosing workflows.

Across the included studies, implementation of MIPD was associated with improved pharmacokinetic target attainment, particularly in studies evaluating vancomycin AUC-guided dosing [16,17,22]. These findings are consistent with the expected role of Bayesian approaches in optimizing drug exposure and supporting individualized dosing. Clinical outcomes were reported in a subset of studies and included reductions in nephrotoxicity, particularly acute kidney injury, as well as measures of clinical effectiveness and bacteremia-related outcomes [7,16,17,18,19]. In addition, some studies reported improvements in dosing management, such as earlier achievement of target exposure and reduced need for dose adjustments [5,15,23]. However, these findings should be interpreted in the context of the predominance of vancomycin studies, where AUC-guided dosing and nephrotoxicity reduction are well-established clinical priorities. As such, the generalizability of these outcomes to other drugs and therapeutic areas remains limited.

Pharmacists played an important role in the implementation and application of MIPD across many of the included studies. Their involvement ranged from consultative services to pharmacist-led dosing programs, contributing to dose individualization, interpretation of Bayesian outputs, and integration of recommendations into clinical care [13,16,17,19]. These findings underscore the importance of clinical expertise in translating model-based recommendations into actionable treatment decisions. In addition, several studies highlighted the integration of MIPD into clinical workflows through software platforms and, in some cases, electronic health record–embedded decision support systems [13,16]. Such integration is likely to be a key factor in facilitating the adoption and sustained use of MIPD in routine practice.

This review has several limitations. First, the number of included studies was relatively small, reflecting the still limited but growing body of published evidence on routine clinical implementation of MIPD. Second, the included studies were heavily concentrated in anti-infective therapy, particularly vancomycin, introducing a therapeutic area bias that may limit the generalizability of the findings to other drugs and clinical settings. Third, there was considerable heterogeneity in study design, populations, MIPD implementation approaches, and reported pharmacokinetic and clinical outcomes, including variation in exposure measures and other PK parameters, which precluded quantitative synthesis, limited direct comparison across studies, and did not support meaningful application of a single formal risk-of-bias framework across all included studies. In addition, exclusion of conference abstracts and proceedings may have limited representation of emerging Bayesian dosing applications not yet reported as full-text studies, although many such records appear to focus on software presentation, validation, or predictive performance rather than routine clinical implementation. Lastly, study selection was performed by a single reviewer, which may have introduced some risk of selection bias, although predefined eligibility criteria supported consistency in study selection.

Future research should focus on expanding the evidence base for MIPD implementation across a broader range of therapeutic areas beyond anti-infectives. There is a need for prospective studies evaluating real-world clinical use, including both pharmacokinetic and clinical outcomes, as well as implementation-related measures such as workflow integration and resource utilization. In addition, more detailed reporting of implementation characteristics, including the role of healthcare professionals, integration into clinical systems, and dosing strategies, would enhance understanding of how MIPD is applied in practice. MIPD may provide the greatest clinical value in settings where conventional TDM alone is insufficient to guide precise dose individualization, particularly for drugs with high pharmacokinetic variability, narrow therapeutic indices, or AUC-based exposure targets. Clinically, this may be especially relevant in scenarios requiring early dose optimization, avoidance of toxicity, or management of patients with variable or changing pharmacokinetics, such as critically ill patients, pediatric populations, and complex anti-infective therapy. In the current literature, this was most evident in vancomycin-focused applications, where Bayesian dosing supported exposure-guided optimization beyond trough-based approaches. This is particularly important because broader implementation may be constrained by practical barriers such as the costs of health care personnel training, information technology support, and software licenses, as well as limited familiarity among clinicians and pharmacists with Bayesian dosing approaches [31,32]. These challenges suggest that wider adoption of MIPD will likely depend not only on stronger clinical evidence but also on institutional support, targeted education, and implementation-ready digital infrastructure [31,33].

In conclusion, this systematic review demonstrates that Bayesian MIPD is being implemented in routine clinical practice, primarily in the context of TDM and predominantly for vancomycin dosing. While the available evidence suggests improvements in pharmacokinetic target attainment and potential clinical benefits, the published literature on real-world implementation remains limited. Greater reporting and evaluation of routine clinical use are needed to fully realize the potential of MIPD across diverse therapeutic areas.

## Figures and Tables

**Table 1:** Characteristics of included studies on clinical use of Bayesian MIPD software in routine practice.

Therapeutic Area	Drug Name	Software Platform	Patient Population	Pharmacist Involvement	Outcome Domains	Conclusion	Country	Publication Year	Ref.
Anti-infectives	Vancomycin	InsightRx	Pediatric (neonates and children)	Pharmacist-led	Implementation and CDS tool use	Integration of a vancomycin CDS tool within the EHR, with clinical pharmacist involvement, enabled successful adoption of MIPD in clinical care.	United States	2020	[13]
Hematopoietic cell transplantation	Busulfan	InsightRx	Pediatric (1–26 years; HCT recipients)	Not reported	First PK target attainment; cAUC target attainment	Early achievement of target exposure improved with the updated busulfan model and Bayesian platform; model-informed dosing and TDM provided advantages over conventional guidelines for achieving target cAUC.	United States	2020	[5]
Anti-infectives	Aminoglycosides, amikacin	Abbottbase; DoseMeRx	Adults (>18 years)	Pharmacist-utilized	Comparison of AUC_24_, Cmax, Cmin (DoseMeRx vs. Abbottbase)	Amikacin dosing and TDM were suboptimal vs. guidelines; the DoseMeRx^®^ model was satisfactory to guide dosing.	Australia	2021	[14]
Anti-infectives	Vancomycin	DoseMeRx	Adults (18–100 years)	Clinical pharmacology service	Primary: Time within target AUC_24_/MIC; Secondary: Time to target attainment	A consultative TDM service facilitated attainment of vancomycin therapeutic targets; further optimization may improve its use.	Australia	2021	[15]
Anti-infectives	Vancomycin	InsightRx	Pediatric (<21 years; cystic fibrosis)	Pharmacist-managed	AUC_24_ target attainment; trough attainment; dosing patterns; extreme troughs; AKI	An MIPD approach within an EHR-integrated CDS tool supported safe vancomycin AUC-guided dosing with high target attainment.	United States	2023	[16]
Anti-infectives	Vancomycin	Not reported	Neonates (Level IV NICU)	Pharmacist-involved	Describing implementation planning, rollout, and software selection	Describes selection, planning, and implementation of Bayesian software for neonatal vancomycin AUC monitoring; informs MIPD tool selection and neonatal considerations.	United States	2023	[6]
Anti-infectives	Vancomycin	DoseMeRx	Adults (≥19 years; suspected/documented MRSA infections)	Pharmacist-managed	Primary: AKI incidence; Secondary: AUC target attainment; AKI timing; SCr increase; dialysis; ICU admission	In patients receiving VPT, Bayesian MIPD resulted in lower AKI rates, higher target attainment, and more usable vancomycin levels vs. first-order AUC dosing.	United States	2023	[17]
Anti-infectives	Vancomycin	InsightRx	Adults (≥18 years; culture-proven Gram-positive infections)	Not reported	Primary: DOOR; AKI; mortality; Secondary: DOOR components; escalation of care; 30-day readmission; ICU LOS; hospital LOS	AUC-guided vancomycin therapy using MIPD improved outcomes in Gram-positive infections, reduced VA-AKI, and allowed earlier AUC assessment with flexible sampling.	United States	2024	[18]
Anti-infectives	Vancomycin	Not explicitly named; InsightRx mentioned in notes	OPAT program	Pharmacist-driven	Primary: Nephrotoxicity; Secondary: 90-day mortality/readmission	Nephrotoxicity was reduced during outpatient vancomycin therapy.	United States	2024	[19]
Transplantation	Mycophenolate mofetil	ISBA	Adults (≥18 years; liver transplant recipients)	Not reported	AUC target attainment; dose adjustment impact	Bayesian dose adjustment during routine follow-up improved MPA exposure and increased target attainment.	France	2025	[20]
Anti-infectives	Vancomycin	DoseMeRx	Adults (≥19 years)	Pharmacist-consulted	Primary: Agreement of AUC_24_ with vs. without steady-state levels; Secondary: Agreement of AUC_24_ with vs. without pre–steady-state levels; category concordance	AUC_24_ estimates showed overall agreement with and without steady-state levels; tighter agreement was observed with steady-state levels.	United States	2025	[21]
Anti-infectives	Vancomycin	NextDose	Adults (≥18 years)	Pharmacist-driven	Primary: AUC target attainment; time to target; Secondary: guideline adherence; nephrotoxicity	Initial AUC_24_ target attainment was low but improved with Bayesian dosing; trough–AUC correlation was modest; guideline adherence was high.	New Zealand	2025	[22]
Anti-infectives	Aminoglycosides, tobramycin	InsightRx	Pediatric (<21 years; cystic fibrosis)	Multidisciplinary (physician and pharmacist)	Primary: Target AUC_24,SS_ attainment; Secondary: starting dose; dose adjustments; TDM frequency; treatment duration	MIPD for tobramycin in pediatric CF enabled early AUC_24_ target attainment with reduced TDM burden and dose adjustments.	United States	2026	[23]
Anti-infectives	Vancomycin	InsightRx	Pediatric	Pharmacist-led	Program implementation (no clinical endpoints)	VIPER, a structured annual education program, was successfully implemented to support a pharmacist-led pediatric vancomycin TDM service, with high pharmacist satisfaction.	United States	2026	[24]
Anti-infectives	Vancomycin	Abbottbase	Pediatric (2 months–18 years; critical care)	Not reported	Exposure comparison of trough- vs. AUC-based TDM and 1- vs. 2-point sampling; TDM and drug effectiveness/toxicity correlation; PK parameters (AUC, trough, CL, Vd); AKI; bacteremia outcomes	AUC-based vancomycin dosing reduced AKI in pediatric patients without compromising efficacy.	South Korea	2026	[7]

AKI, acute kidney injury; AUC_24_, area under the concentration–time curve over 24 h; CDS, clinical decision support; cAUC, cumulative area under the concentration–time curve; CL, clearance; DOOR, desirability of outcome ranking; EHR, electronic health record; HCT, hematopoietic cell transplantation; ICU, intensive care unit; ISBA, ImmunoSuppressant Bayesian Dose Adjustment; LOS, length of stay; MIPD, model-informed precision dosing; MRSA, methicillin-resistant *Staphylococcus aureus*; NICU, neonatal intensive care unit; OPAT, outpatient parenteral antimicrobial therapy; PK, pharmacokinetic; SCr, serum creatinine; TDM, therapeutic drug monitoring; Vd, volume of distribution.

## Data Availability

The search strategies are provided in the Supplementary Materials. No analytic code was generated for this review.

## Supplementary Materials

The following supporting information can be downloaded at: https://www.mdpi.com/article/10.3390/jcm15103838/s1, Supplementary S1: PRISMA checklist; Supplementary S2: Search strategy. Reference [12] is cited in the Supplementary Materials.
